# Regional context and realization of fertility intentions: the role of the urban context

**DOI:** 10.1080/00343404.2019.1599843

**Published:** 2019-04-29

**Authors:** Bernhard Riederer, Isabella Buber-Ennser

**Affiliations:** a Austrian Academy of Sciences, Wittgenstein Centre (IIASA, VID/ÖAW, WU), VID/ÖAW, Vienna, Austria; and Department of Sociology, University of Vienna, Vienna, Austria.; b Austrian Academy of Sciences, Wittgenstein Centre (IIASA, VID/ÖAW, WU), VID/ÖAW, Vienna, Austria. isabella.buber@oeaw.ac.at

**Keywords:** fertility intentions, urban–rural differences, Generations and Gender Survey, J13, P25, R00, Y80

## Abstract

Despite regional variation in fertility, rural–urban differences in the realization of fertility intentions have not been addressed in previous research. This paper analyzes the realization with data from 11 European countries, employing binomial and multinomial logistic regression models, decomposition analyses, and examining the role of contextual factors. The results demonstrate that realization is lower in urban than in rural regions. In cities, postponement of childbearing is much more common. This can be partly explained by differences in characteristics (e.g., age, partnership status) of inhabitants who intend to have a(nother) child. Furthermore, contextual factors such as educational and economic opportunities play a role.

## INTRODUCTION

Low levels of fertility in Europe have drawn social scientists’ attention to the driving forces behind it and the possible consequences for societies at large (Lutz, 2009; van de Kaa, 1987). In this context, the study of fertility intentions has gained importance, with intentions analyzed from different perspectives and in different country contexts (e.g., Billari, Philipov, & Testa, 2009; Hagewen & Morgan, 2005). Apart from intended family size and the desired number of children in a long-term perspective, a branch of the literature explores short-term fertility intentions, on the one hand, and their realization, on the other (Régnier-Loilier & Vignoli, 2011; Spéder & Kapitány, 2009). Despite regional variations and rural–urban differences in fertility (Kulu & Boyle, 2009; Kulu, Vikat, & Andersson, 2007), to our knowledge this aspect has not been addressed in detail in the realm of realizing fertility intentions. Life circumstances in cities may be of particular importance for the question whether or not individuals can realize their childbearing intentions.

In general, capitals are of great relevance as major cities often play a key role in explaining the spread of new trends and changes in social behaviour. Cities are centres of economic activity (Scott & Storper, 2015). In a historic perspective, urbanization and demographic transitions seem to be highly interrelated (Bocquier & Costa, 2015; Jaffe, 1942; Sharlin, 1986). In the last decades, cities were at the forefront of the structural change of industries and the growth of information and service sectors (Kazepov, 2005; Scott & Storper, 2015; Storper, 2013). The respective changes in the labour market contributed to less stable careers complicating life and family planning. Historically, fertility decreases started earlier and went on faster in cities than in rural regions. Capitals and larger cities usually have lower fertility rates than rural areas (e.g., de Beer & Deerenberg, 2007; Hank, 2001, 2002; Kulu, 2013; Kulu et al., 2007; Kulu & Washbrook, 2014). Though fertility differentials between urban and rural areas are smaller than in the past, they still exist (Kulu et al., 2007). By focusing on realizing the intention to have a child within three years in cities and rural areas, the current study strives to obtain further insights in fertility behaviour with a regional perspective.

We contribute to the literature by differentiation between urban and rural regions. Studies revealed that sociodemographic characteristics and competing intentions in other life domains affect the realization of short-term fertility intentions (e.g., Berrington, 2004; Kapitány & Spéder, 2012; Morgan & Rackin, 2010; Régnier-Loilier & Vignoli, 2011; Spéder & Kapitány, 2009). But even if differences in regions were studied (e.g., Northern versus Southern Italy or Eastern versus Western Germany) (Kuhnt & Trappe, 2016; Mencarini, Vignoli, & Gottard, 2015; Rinesi, Pinnelli, Prati, Castagnaro, & Iaccarino, 2011), urban–rural comparisons in the realization of fertility intentions have not been conducted. To the best of our knowledge, only Mencarini et al. (2015) included municipality size, distinguishing between big, medium and small communities. Not a single study, however, elaborated on urban–rural differences in detail.

## REALIZATION OF FERTILITY INTENTIONS AND REGIONAL DIFFERENCES

Scholars addressing fertility intentions and their realization mainly refer to the theory of planned behaviour (TPB) (Ajzen & Fishbein, 2005) or to the traits–desires–intentions–behaviour theory (Miller, Severy, & Pasta, 2004). According to the TPB, intentions depend on attitudes, social norms, perceived behavioural control and background factors. The latter theory underlines the importance of proceptive behaviour. In both theories, enablers and restrictions are relevant for realizing fertility intentions.

In the present paper, the regional context is mainly regarded as enabler or restriction affecting the realization of existing childbearing intentions. As the above-mentioned theories do not discuss the regional context, we draw upon the literature on actual fertility differentials to fill this gap. This branch of research generally identifies contextual as well as compositional factors, and specifically distinguishes three different reasons why fertility differences between regions exist: (1) regional opportunity structures, (2) local patterns of social interactions/cultural norms and (3) distribution of individual characteristics (Hank, 2002; Kulu & Washbrook, 2014; Trovato & Grindstaff, 1980). In our view, these theoretical arguments can also be applied to realizing childbearing intentions.

Countries as well as urban and rural regions within countries usually differ in several aspects that are relevant to reproductive behaviour and fertility. Opportunity structures (see regional opportunity structures above) might affect the chances of realization as they influence the ability to provide the appropriate environment seen as a prerequisite for parenthood. Childcare facilities, female employment and costs of living are crucial for family formation. On the one hand, urban environments usually offer more possibilities regarding formal childcare and thus improve the reconciliation of family and professional life (Kravdal, 1996; Verwiebe, Troger, & Riederer, 2014). This may enhance the realization of intentions. On the other hand, more educational and labour market opportunities in metropolitan areas might compete with family plans and childbearing. In addition, living costs are higher in cities. This may foster the postponement of fertility.

Norms and attitudes towards parenthood also differ between countries or regions (see local patterns of social interactions/cultural norms). If, for instance, traditional family views are stronger in rural than in urban areas (Carter & Borch, 2005; Glenn & Hill, 1977), parenthood might be more relevant for individuals’ life plans in rural settings, which might increase the chances of realizing fertility plans.

Furthermore, characteristics of individuals living in cities and in the countryside likely differ from each other (Hank, 2002) (see the distribution of individual characteristics). In urban areas people are usually higher educated (Spielauer, Schwarz, Städtner, & Schmid, 2003). As longer periods of education encourage postponement of parenthood, higher shares of highly educated in cities, that is, a different composition of urban and rural populations, argue again for lower realization rates in cities than in rural areas.

Overall, we hypothesize that the realization of childbearing intentions is lower in urban than in rural areas. Most of the discussed characteristics of cities support our hypothesis (educational and labour market opportunities, values, safety). Only the availability of formal childcare argues against it. However, the availability of informal childcare might be higher in rural contexts. Urban life usually offers many alternatives that might compete with childbearing and childrearing. It may thus foster a postponement of childbearing to later periods or abandonment of earlier intentions.

In terms of the above-mentioned general distinction in contextual and compositional factors, regional opportunity structures are contextual ones, whereas the distribution of individual characteristics refers to the composition of rural and urban populations. Cultural norms are related to both context and composition: norms and values might be understood as contextual factors. Values, however, result in different attitudes of people (i.e., population characteristics).

Effects of context and composition can hardly be disentangled from each other when it comes to rural–urban differences in fertility. First, opportunities, values and population composition are not independent from each other. For instance, young people often move to cities for educational reasons (opportunities). The higher educated have usually fewer traditional attitudes and want to pursue careers (values). Thus, higher shares of highly educated may lead to lower realization rates in cities (composition).

Second, context affects composition via selective migration (Frey & Kobrin, 1982). As cities with higher rates of crime and less open green space than rural areas are usually not perceived as ideal places to raise children (Kulu & Vikat, 2007), many people move from cities to rural areas shortly before or after the birth of a child (Kulu & Boyle, 2009; Mulder & Wagner, 2001). Nevertheless, we will account for the role of composition in parts of our empirical analyses (see below).

For our analyses of rural–urban differences in realization of fertility intentions, we use available data for 11 European countries. Family policies, labour market structures and gender norms vary substantially across Europe (Matysiak & Węziak-Białowolska, 2016). Such country-specific conditions are usually believed to affect childbearing behaviour. Although cross-country comparisons of realization of fertility intentions have already been conducted (e.g., Kapitány & Spéder, 2012; Régnier-Loilier & Vignoli, 2011), evidence is still sparse. Existing studies reveal substantial variation across European countries. In particular, research suggests that post-communist societies have lower realization rates due to the character and pace of social change after 1989–90, discontinuity of political support and resulting feelings of anomie (Kapitány & Spéder, 2012; Spéder & Kapitány, 2014). We thus assume realization to be higher in Western European countries than in Eastern Europe. As a consequence, we differentiate between continental Western Europe and Eastern Europe in our analyses. As we cannot identify any reasons in the literature why urban–rural differences in realization should vary across countries, however, we suppose that our hypothesis on urban–rural differences will hold for both regions under investigation (and irrespective of differing national backgrounds).

## DATA, VARIABLES AND ANALYTICAL STRATEGY

The current study is based on the Generations and Gender Survey (GGS) – a panel study with detailed information on family formation and fertility^1^ – and includes 11 countries: four in continental Western Europe (Austria, France, Germany and the Netherlands) and seven (former socialist) Eastern European countries (Bulgaria, Czechia, Georgia, Hungary, Lithuania, Poland and Russia).^2^ Table 1 gives national sample sizes.

**Table 1.** Generations and Gender Survey (GGS) respondents aged 18–45 years.

Sample	Time 1	Longitudinal	Longitudinal with fertility intentions at time 1		
	Total	Total	Total	Rural	Urban
*Western Europe*					
Austria	4994	3908	1110	439	671
France	4870	3191	836	383	453
Germany	4789	1389	336	69	267
Netherlands	4141	3073	540	264	276
	18,794	11,561	2822	1155	1667
*Eastern Europe*					
Bulgaria	7986	5680	1704	458	1246
Czechia	5289	1534	377	114	263
Georgia	5315	4403	1685	739	946
Hungary	6359	4990	2421	1567	854
Lithuania	4972	1037	247	101	146
Poland	8414	4726	1147	385	762
Russia	5613	3757	926	566	360
	43,948	26,127	8507	3930	4577
Total	62,742	37,688	11,329	5085	6244

Source: Generations and Gender Survey (GGS) waves 1 and 2.

We study the realization of short-term fertility intentions by analyzing whether or not those who wanted a(nother) child within three years at wave 1 have realized their intentions until wave 2. In line with previous studies (Kapitány & Spéder, 2012; Spéder & Kapitány, 2009), we further differentiate between those still wanting a child (postponement) and those who do not any longer want a(nother) child (abandonment) (Table 2), whenever this information is available. Binomial and multinomial logistic regression models are carried out to estimate average marginal effects. They represent the average effect of a variable on the probability of realization (postponement or abandonment, respectively) and are comparable across different models (cf. Best & Wolf, 2012). Positive coefficients indicate that the corresponding group more often realized (postponed/abandoned) short-term fertility intentions; negative coefficients indicate that these were less often realized (postponed/abandoned).

**Table 2.** Definition of fertility intentions and outcomes.

Types	Fertility intention and outcome		
	Intended to have a child within three years at time 1	Birth of a child between times 1 and 2	Intend to have a child at time 2
Realization	Yes	Yes	–
Postponement	Yes	No	Yes
Abandonment	Yes	No	No

Source: Kapitány and Spéder (2012, p. 606); adapted by the authors.

The explanatory variable of interest is regional context. Based on the Organisation for Economic Co-operation and Development’s (OECD) regional typology (2011), we distinguish between rural regions and urban areas. This typology has been implemented in the GGS by the majority of countries. The OECD applies criteria of population density and population size of urban (regional) centres. France, Germany and Lithuania originally used a more detailed classification of population size, while the type of settlement is based on addresses/km^2^ in the Netherlands. In the present study, settlements with at least 50,000 inhabitants as well as those with at least 1500 addresses/km^2^ were classified as urban (for details of the classification for each country, see Table A1 in Appendix A in the supplemental data online).^3^

Various sociodemographic characteristics are considered as control variables: (1) gender; (2) age (years; 18–24, 25–29, 30–34, 35–45); (3) partner status (co-resident, living apart together (LAT), no partner); (4) parity (childless, one child, two children, three and more children); (5) education (International Standard Classification of Education – ISCED: 0–2, 3–4 and 5–6). Measures refer to wave 1. The existing literature has repeatedly shown that these variables affect the realization of fertility intentions (Régnier-Loilier & Vignoli, 2011; Spéder & Kapitány, 2009; Spéder & Kapitány, 2014). In addition, we control for country of residence and the time span (months) between the two interviews (34–48, 49–60, 61–72, 73–80).

To capture context factors, we consider indicators for (a) childcare opportunities, (b) female employment opportunities, (c) educational and labour market opportunities, (d) financial affordability of living and (e) childbearing norms. Owing to the availability of data, we selected for these five broad aspects the following indicators: (a) share of parents with a child less than three years of age using formal childcare, (b1) share of employed mothers, (b2) share of fulltime employed mothers, (c1) share of highly educated persons, (c2) share of high skilled professional occupations, (d) share of persons reporting difficulties to make ends meet, (e1) share of childless women aged 40–45 years and (e2) share of respondents agreeing to the statement ‘A woman has to have children in order to be fulfilled’ (for details, see Appendix A in the supplemental data online).

Our analytical strategy comprises several steps. First, descriptive analyses depict differences in realization, postponement and abandonment between rural and urban areas.

Second, the impact of regional context on realization is explored in multiple logistic regressions. Therein, we follow a stepwise hierarchical model build up: a basic model (M1) only includes our main explanatory variable (regional context). Model M2 adds country and time span as control variables and model M3 the sociodemographic characteristics mentioned above. The method developed by Karlson, Holm, and Breen (2012) is employed to prove whether adding explanatory variables changes the difference between urban and rural regions (KHB test).^4^ If so, it can be assumed that the added variables are responsible for differences between urban and rural regions. This method is also applied in multinomial models (realization/postponement/abandonment).^5^ These analyses are carried out for the pooled sample, as well as for Western and Eastern Europe separately, to find out an overall effect and to explore possible differences by regions.

Third, the role of contextual factors is explored. We analyze whether urban–rural differences in realization and postponement are smaller if we control for context variables on the regional level (model M4). In addition, we employ multilevel modelling to assess how much of the regional variation can be explained by contextual characteristics. In these models, contextual variables refer to 22 regions (one urban and one rural region in each of the 11 countries).^6^

Finally, decomposition analyses examine the impact of differences in characteristics of urban and rural populations on urban–rural differences in realization and postponement of childbearing intentions. Therefore, we employ methods proposed by Fairlie (2005), Jann (2006) and Sinning, Hahn, and Bauer (2008) and calculate the contribution of compositional factors. Analyses for single countries are additionally provided in Appendix A in the supplemental data online (and will be occasionally described in the notes).

Descriptive analyses are based on panel respondents intending to have children within the next three years at wave 1 (*N* = 11,329). Owing to missing values in controls and unknown fertility intentions at wave 2, multinomial models for realization/postponement/abandonment are restricted to a slightly smaller sample (*N* = 10,137) (see Table A2 in Appendix A in the supplemental data online). Contextual factors were computed using GGS data of wave 1, including all respondents aged 18–45 years (*N* = 62,742), to guarantee that differences in context factors refer to the same classification of urban and rural regions.

## RESULTS

### Regional differences in realization, postponement and abandonment

**Figure 1.**
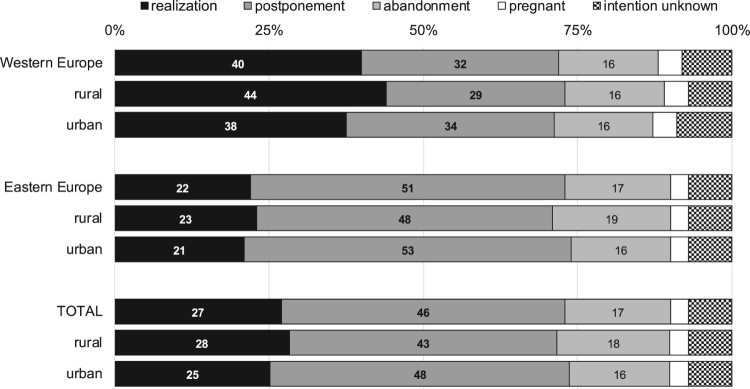
Fertility outcome and fertility intentions by country cluster. Note: ^(^*^)^*p* ≤ 0.1; *p ≤ 0.05; **p ≤ 0.01; ***p ≤ 0.001. Sources: Generations and Gender Survey (GGS) waves 1 and 2; panel respondents intending a child within three years in wave 1 (*N* = 11,329).

Overall, short-term intentions were more often realized in Western than in Eastern Europe: four in 10 Western Europeans but only two in 10 Eastern Europeans intending a child within the next three years at wave 1 had a new-born child at wave 2 (Figure 1). This is mainly due to higher postponement in Eastern Europe. Abandonment is less common in both macro-regions (16% versus 17%).

Short-term fertility intentions were less often realized in the urban than in rural areas (28% and 25%) (Figure 1). This difference is larger in Western European countries, amounting to 6 percentage points, than in Eastern ones (2 percentage points). In Western and Eastern Europe, individuals more often postpone their plans in urban areas. Overall, 48% of respondents from urban regions and 43% of respondents from rural regions did not realize their intentions but still wanted to have a(nother) child. Country-specific analyses (see Figure A1 in Appendix A in the supplemental data online) reveal urban–rural differences in realization in all Western countries and in the majority of Eastern countries. Postponement is more frequent in urban than in rural regions in 10 of the 11 countries under study.

Our basic regression model confirms lower realization in capitals than in other regions of the countries (Table 3, panel a, model M1). This regional difference remains statistically significant in multivariate models including country of residence and time span between the two interviews (model M2) and when further controlling for sociodemographic characteristics (model M3) (see Table A3 in Appendix A in the supplemental data online for results on control variables). In addition, analyses by single countries reveal regional differences for almost every country under study (see Figure A2 online).^7^ Multinomial regressions (which distinguish between realization, postponement and abandonment) once more reveal that people living in urban areas postpone more often than those from rural regions (Table 3, panel b, models M1–M3; for details on controls, see Table A4 online). According to additionally applied KHB tests, control variables hardly account for regional differences in realization, postponement and abandonment (Table 3). In other words, results for the pooled sample suggest that differences in sociodemographic characteristics between urban and rural regions are not responsible for lower realization and higher postponement in urban regions. Results of multilevel models with differing model specifications confirm these conclusions (see Table A5 online).

**Table 3.** Regional differences in realization, postponement and abandonment of childbearing intentions (average marginal effects).

	Model M1		Model M2		Model M3		Model M4	
	*b*	AME	*b*	AME	*b*	AME	*b*	AME
(a) *Binomial logistic regression: regional differences in realization of intentions*								
*Realization (dichotomous)*								
Rural regions (reference)	0	0	0	0	0	0	0	0
Urban regions	−.12**	−.02**	−.14**^a^	−.03**	−.17***^a^	−.03***	.00^a^	.00
Cragg–Uhler *R*^2^	.00		.08		.19		.19	
*N*	11,319		11,319		11,319		11,319	
(b) *Multinomial regression: differences in realization, postponement, and abandonment of intentions*								
*Realized*								
Rural regions (reference)	0	0	0	0	0	0	0	0
Urban regions	−.18***	−.03**	−.21***^b^	−.03**	−.23***^b^	−.03***	.07^e^	.01
*Postponed*								
Rural regions (reference)	0	0	0	0	0	0	0	0
Urban regions	0	.05***	0	.05***	0	.05***	0	−.01
*Abandoned*								
Rural regions (reference)	0	0	0	0	0	0	0	
Urban regions	−.21***	−.02**	−.25***^c^	−.03***	−.23***	−.02*^d^	.01^d^	.00
Cragg–Uhler *R*^2^	.00		.09		.35		.35	
*N*	10,137		10,137		10,137		10,137	

Notes: For details, see Tables A3 and A4 in Appendix A in the supplemental data online. AME, average marginal effects. (*)*p* ≤ .1; *p ≤ .05; **p ≤ .01; ***p ≤ .001.

^a^KHB test indicates no significant difference in realization versus non-realization (*p* > 0.10) between models M1 and M2, M2 and M3, or M3 and M4.

^b^KHB test indicates no significant difference in realization versus postponement (*p* > 0.10) between models M1 and M2 or M2 and M3.

^c^KHB test indicates significant difference in postponement versus abandonment (*p* ≤ 0.05) between models M1 and M2.

^d^KHB test indicates no significant difference in postponement versus abandonment (*p* > 0.10) between models M2 and M3 as well as between M3 and M4.

^e^KHB test indicates an almost significant difference in realization versus postponement (*p* ≤ 0.10) between models M3 and M4.

Sources: Generations and Gender Survey (GGS) waves 1 and 2; panel respondents intending a child within three years in wave 1.

**Figure 2.**
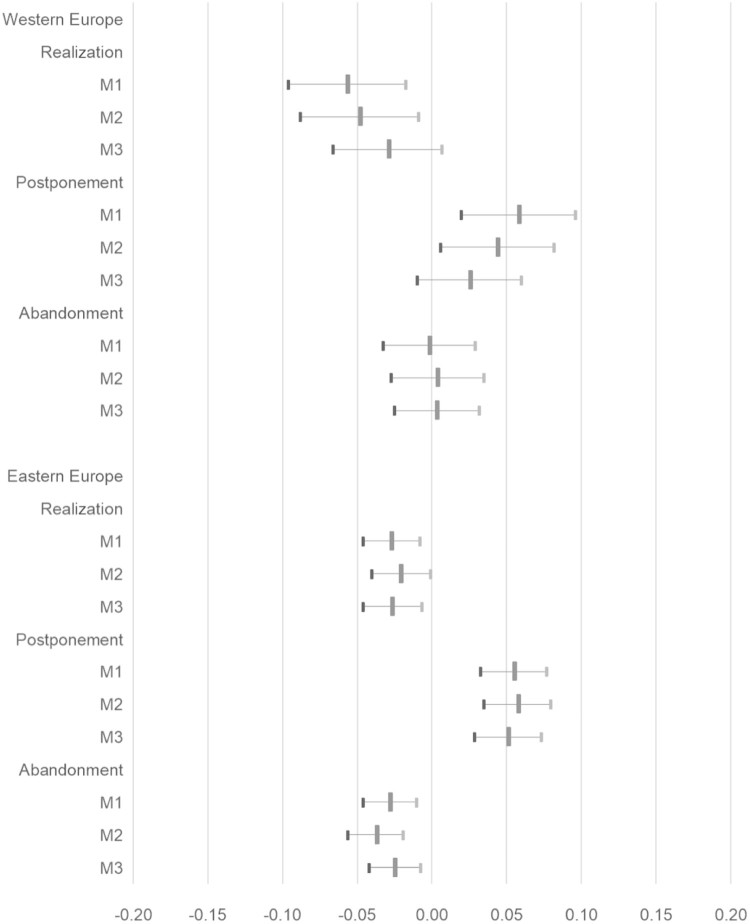
Urban–rural differences in realization, postponement and abandonment of fertility intentions by country cluster (average marginal effects). Note: Data are average marginal effects (AME) and corresponding 95% confidence intervals resulting from multinomial logistic regression models (model M1 without controls, models M2 and M3 including controls; for details, see the methods section). Sources: Generations and Gender Survey (GGS) waves 1 and 2; panel respondents intending a child within three years in wave 1.

Analyses distinguishing between Western and Eastern Europe, however, indicate an interesting pattern: Urban–rural differences are merely affected by control variables in Eastern Europe, but are smaller – both for realization and postponement – in Western Europe if sociodemographic variables are included (model M1 versus M3 in Figure 2). In addition, lower realization in urban regions in Western Europe leads to higher postponement but does not affect abandonment of intentions. In Eastern Europe, higher postponement in urban regions reflects not only lower realization but also lower abandonment of childbearing intentions (Figure 2).

### Reasons behind regional differences in realization: on contextual and compositional effects

In the theoretical section, we argued that contextual characteristics may be responsible for urban–rural differences in realization of fertility intentions.^8^ In both Western and Eastern Europe, we observe (1) higher usage rates of childcare, (2) larger shares of mothers in fulltime employment, (3) larger shares of highly educated people, (4) larger shares of high skilled professional occupations, (5) larger shares of childless women age 40–45 years and (6) lower shares of people agreeing that women need motherhood to be fulfilled in urban than in rural regions (Table 4). However, overall maternal employment (including full- and part-time) is higher in urban than in rural regions only in the East. Furthermore, economic difficulties are reported less often in urban than in rural regions in Eastern but not in Western Europe.^9^

**Table 4.** Urban–rural differences in context variables.

Shares are given in %; differences in percentage points	Western Europe		Eastern Europe	
	Rural	Urban (±)	Rural	Urban (±)
*Childcare opportunities*				
Use of childcare among children less than 3 years of age	43	+2	25	+4
*Maternal employment*				
Mothers employed (age 25–45 years)	66	−4	60	+5
Mothers fulltime employed (age 25–45 years)	25	+2	52	+8
*Educational and labour market opportunities*				
Share of highly educated (ISCED 5–6) (age 25–45 years)	24	+10	19	+18
Share of high skilled professional occupations (ISCO 1–3) (age 25–45 years)	41	+11	26	+18
*Economic situation*				
Share of people with difficulties making ends meet (age 18–45 years)	39	+1	72	−6
*Norms/family views*				
Share of childless women (age 40–45 years)	11	+8	7	+3
Share agreeing that women need child(ren) to be fulfilled (age 18–45 years)	41	−7	70	−9

Note: ISCO, International Standard Classification of Occupations.

Source: Generations and Gender Survey (GGS) wave 1 (62,742 respondents aged 18–45 years).

Our results show that context characteristics contribute to regional differences in realization and postponement: Urban–rural differences in realization and postponement are no longer statistically significant when contextual variables are added (Table 3, model M4). KHB tests, however, cannot confirm throughout that coefficients in model M4 differ from those in model M3. According to additional multilevel analyses (see Table A9 in Appendix A in the supplemental data online), contextual factors account for approximately 40–45% of the regional variation in realization and postponement but cannot explain regional differences in abandonment.

Regarding single contextual factors, our main findings can be summarized as follows. Higher rates of childcare usage and maternal employment go along with higher realization of intentions, less postponement of childbearing and higher abandonment. Enhanced educational and labour market opportunities seem to lead to lower realization and more postponement. The higher the share of people with difficulties to make ends meet, the less likely are both realization and postponement, and the more likely is abandonment of fertility intentions. Findings regarding norms (childlessness, relevance of motherhood) are not straightforward (for details, see Tables A8 and A9 in Appendix A in the supplemental data online).

Finally, we turn to another potential source for regional differences in realization and postponement of childbearing intentions, namely differences between urban and rural populations. By estimating a counterfactual (‘what if’) distribution, a decomposition analysis allows one to assess whether differences in realization between urban and rural areas can be attributed to the composition of individuals in the respective regions. Results indicate that different compositions explain a substantial part of regional differences in realization and postponement in Western but not in Eastern Europe (Table 5). This is in line with findings on urban–rural differences in Figure 2 described above. In Western Europe, about half the urban–rural difference in realization (3.2 of 5.8 percentage points) and in postponement (2.4 of 5.8 percentage points) is explained by differences in urban and rural populations (Table 5).^10^ In particular, the larger shares of singles with childbearing intentions and of persons in advanced reproductive age (i.e., 35–45 years) in urban regions result in lower realization rates (see Table A10 in Appendix A in the supplemental data online). Higher postponement in Western Europe's urban regions than in its rural regions is mainly driven by larger shares of singles and LAT couples as well as lower proportions of parents with two or more children wanting (additional) children in urban areas.

**Table 5.** Results of decomposition analyses for country clusters (model M3).

(a) *Realization (dichotomous)*	Probability of realization		Difference in realization	Explained by composition
	Rural	Urban		
Western Europe	.438	.380	.058	.032
Eastern Europe	.232	.209	.023	−.006
**(b) *Postponement (dichotomous)***	**Probability of postponement**		**Difference in postponement**	**Explained by composition**
	**Rural**	**Urban**		
Western Europe	.326	.384	−.058	−.024
Eastern Europe	.532	.587	−.055	−.011
**(c) *Realization, postponement, abandonment (ordinal)***			**Heterogeneity measure**	**Explained by composition**
Western Europe			.067	.026
Eastern Europe			.074	−.032

Note: For detailed results of decompositions (a) and (b), see Table A6 in Appendix A in the supplemental data online. Decomposition analyses refer to model M3 without time span and country. Our conclusions are not altered if these variables are additionally included (but the sum of effects in Table A6 would not correspond to coefficients in Table 4). Decomposition (c) understands realization, postponement and abandonment as ordinal sequence (birth occurred/child wanted/no child wanted).

Sources: Generations and Gender Survey (GGS) waves 1 and 2; panel respondents intending a child within three years in wave 1.

Detailed results of decomposition analyses additionally reveal noteworthy differences between Western and Eastern Europe.^11^ First, the higher share of highly educated in urban regions (ISCED 5–6) fosters a realization in cities in both macro-regions.^12^ At the same time, it fosters postponement in urban areas in Eastern Europe while it counteracts postponement in cities in Western Europe. This suggest that, in Eastern Europe, abandonment seems to be lower in cities due to educational differences in urban and rural populations.

Second, in the West singles intending a child in the near future are more often found in urban regions than in rural settings, which contributes to lower realization and higher postponement in urban than in rural areas (see Table A10 in Appendix A in the supplemental data online). In contrast, in Eastern Europe, singles with childbearing intentions are less frequent in urban than in rural areas (and this compositional difference is counteracting lower realization and higher postponement in cities).

## DISCUSSION

The present paper analyzed the role of regional context for the realization of short-term fertility intentions. Although research has repeatedly demonstrated regional variation and rural–urban differences in fertility (e.g., Kulu & Boyle, 2009), this issue has – to our knowledge – not been addressed before in the realm of realizing fertility intentions. Taken together, our findings demonstrate the relevance of urban areas in this respect, the regional context might be regarded as an enabling or restricting factor for realizing fertility intentions (Ajzen & Fishbein, 2005; Miller et al., 2004).

First, realization was lower in urban than in rural regions in Europe. Differences have been shown in descriptive and various multiple regression analyses. In addition, postponement of intentions turned out to be more common in urban regions. Our main hypotheses on regional differences have thus been confirmed.

Second, decomposition methods demonstrated that urban–rural differences in realization and postponement are partly explained by differences between urban and rural populations. At least in Western Europe, lower realization and higher postponement in cities than in rural areas is driven by larger shares of persons in advanced reproductive age, singles and LAT couples who intend to have a(nother) child in the near future.

Third, our study suggests that contextual factors are relevant for urban–rural differences in realization of fertility intentions. For instance, greater economic opportunities in cities seem to foster postponement while a culture supportive of female employment (higher childcare rates, higher maternal employment) seems to facilitate realization and to promote abandonment of childbearing intentions at the same time.

Finally, we have also gained interesting insights in differences between Western and Eastern Europe. In line with differences in context characteristics (e.g., a higher share of persons with economic difficulties, lower levels of trust in others) and the literature (e.g., Spéder & Kapitány, 2014), we found lower realization rates in the East. While compositions of urban and rural populations could explain a relevant part of urban–rural differences in realization in the West, differences in context may be the main reason for higher postponement in Eastern European cities.

Our study extended prior research in an important way but obviously had limitations as well. First, the differentiation between urban and rural regions was rather crude due to data limitations. We were not able to differentiate further within urban regions with 50,000 or more inhabitants or between cities and suburbs as a residential context (Kulu & Boyle, 2009).^13^

Second, aspects that could not be included due to unavailability of data in the GGS (e.g., housing conditions or informal childcare) might affect the realization of fertility intentions (Aassve, Meroni, & Pronzato, 2012; Vignoli, Rinesi, & Mussino, 2013). Future studies using richer data are thus needed to further deepen insights on regional differences in realization.

Overall, differences in realization across countries were larger than differences between rural and urban regions within countries. National policies and cultural aspects (e.g., norms, values) are certainly important. In addition, the behaviour of individuals who decided to have a child may be very similar, regardless of urban or rural context. Nevertheless, urban areas matter for the realization of fertility intentions, in particular via more frequent postponement. Regional differences within countries are often neglected in family research and should be taken into consideration to a larger degree. As more and more people live in cities, the question whether urban context enables or restricts the realization of childbearing intentions will gain in importance in the future.

## Supplementary Material

Supplemental Material

## NOTES

1. For the data from the GGS, see www.ggp-i.org/data/.

2. GGS panel data are available for 13 countries. Two had to be excluded, namely Australia (due to missing childbearing intentions within the next three years) and Italy (several reasons: missing parity for persons below age 25 years, the questionnaire does not allow one to generate several contextual variables used in our analysis, etc.).

3. Alternative specifications for urban regions for the countries with detailed information on settlement (France, Germany, Lithuania and the Netherlands) with the threshold being 100,000 or 10,000 inhabitants lead to similar coefficients for urban regions (as well as for the control variables). All conclusions remain the same.

4. The KHB test applies the principles of the well-known Sobel test often used in proving the mediation effects to logit models. A specific method is needed as the variance of the underlying latent variable will differ between models and thus the change in the coefficient of a variable cannot be straightforwardly attributed to the inclusion of further variables (Karlson et al., 2012).

5. We present only models including country dummies (fixed effects). Additional multilevel models are presented in Appendix A in the supplemental data online. Table A5 online gives sensitivity analyses with alternative model specifications (two-level random intercept and random coefficient models as well as a three-level random intercept model).

6. In addition, correlations between urban–rural differences in contextual factors and probabilities of (1) realization as well as (2) postponement on the country level are shown in Table A7 in Appendix A in the supplemental data online.

7. Nevertheless, as sample sizes for single countries are usually small, confidence intervals are often large.

8. We focus on urban–rural differences. Western and Eastern Europe generally vary in many regional characteristics. For example, GGS data show that trust in other people is higher in Western than in Eastern Europe (e.g., 37% trust in others in Germany but only 18% in Georgia). Furthermore, in Eastern Europe the proportion of people with economic difficulties is large and maternal employment is usually fulltime (while often part-time in many Western countries). These differences may contribute to explain why realization is lower in the East than in the West of the continent.

9. In one case (Austria), economic difficulties are even more frequent in cities. Detailed figures for single countries are presented in Table A6 in Appendix A in the supplemental data online.

10. The decompositions refer to our specific sample, that is, persons intending a child within three years, and not to the general population.

11. Decompositions for single countries and detailed results of decomposition analyses for selected countries are provided in Tables A11 and A12 in Appendix A in the supplemental data online.

12. Overall, both higher and lower education increase chances of realization (see Tables A3 and A4 in Appendix A in the supplemental data online).

13. A further differentiation within urban regions (e.g., medium-sized cities, large cities, metropolitan cities) would allow one to observe differences in realization in more detail, but is not possible for many countries with GGS data. More detailed results for Austria, for instance, show that realization is lower in the capital (Vienna) than in other urban regions.
