# Supplementary material for: Regional context and realization of fertility intentions: the role of the urban context
Source: Reg Stud. 2019 Apr 29;53(12):1669–79. doi: 10.1080/00343404.2019.1599843 (PMC6824246; doi:10.1080/00343404.2019.1599843)
Supplement: Supplemental Material [file CRES_A_1599843_SM4944.pdf]

Appendix A

Riederer & Buber-Ennser

Regional context and realization of fertility intentions: The role of the urban context

This appendix provides details regarding the definition of urban and rural regions (Table A1), samples sizes for multivariate analyses (Table A2), measurement of contextual variables (text below), models discussed in the main text (Tables A3, A4, and A10), additional insights gained from multilevel models (Tables A5), models including contextual variables (Tables A7-A9), and analyses for single countries (Figures A1 and A2; Tables A6, A11, and A12).

Contextual variables

To capture context factors we consider indicators for (a) childcare opportunities, (b) female employment opportunities, (c) educational and labour market opportunities, (d) financial affordability of living, and (e) family norms and values. These indicators are calculated by using data on 62,742 men and women aged 25-45 included in GGS wave 1. As mentioned in the main text, other sources than the GGS did not allow to assess the differences in context factors between urban and rural regions in an appropriate way. Due to availability of data, we selected for these five broad aspects the following indicators:

(a) Share of parents with a child below age 3 using formal childcare: Share of respondents with a child below age 3 in the household who get regular help from a day care center, a nursery or pre-school, a self-organized childcare group, a babysitter, or other institutional or paid arrangements. Numbers refer to parents aged 17-45 years.

(b1) Share of employed mothers: Share of mothers aged 25-45 years who are employed.

(b2) Share of fulltime employed mothers: Share of mothers aged 25-45 years who are employed full-time.

(c1) Share of highly educated persons. Share of highly educated persons (ISCED 5-6) in the age-span persons 25-45 years with high education.

(c2) Share of economic active people in professional, managerial, or technical positions: Share of persons aged 25-45 years who are employed in an economic branch classified as ISCO 1 (professional), ISCO 2(managerial) or ISCO3 (technical position).

(d) Share of persons reporting difficulties to make ends meet: The exact wording of the question was: "Thinking of your household's total monthly income, is your household able to make ends meet?" Possible answers ("With great difficulty", "with difficulty", "with some difficulty", "fairly easily", "easily", and "very easily") were dichotomised into "difficulties" (the first three groups) and "no difficulties" (the last three groups). Numbers refer to women and men aged 17-45 years.

(e1) Share of childless women aged 40-45: Share of women aged 40-45 years with no natural children.

(e2) Share of respondents agreeing to the statement "A woman has to have children in order to be fulfilled". Possible answers ("Strongly agree", "agree", "neither agree nor disagree", "disagree", and "strongly disagree") were dichotomised into "agreement" (the first two groups) and "others" (the last three groups). Numbers refer to women and men aged 17-45 years.

Some indicators are missing for single countries due to unavailability of necessary information (ISCO classification in Germany, part-time employment in Poland, making ends meet and attitudes towards children in the Netherlands). Main text Table 6 provides the number of countries the calculated indicator is based on (right column).

Effects of controls on realization, postponement, and abandonment

Tables A3 and A4 provide details regarding models discussed in the main text (Table 3). In the following, we shortly discuss effects of control variables on realization, postponement, and abandonment of childbearing intentions.

As expected, realization rates are higher in Western than in Eastern European countries. In addition, postponement seems to be less frequent in Western than in (most) Eastern European countries. The timespan between the two interviews hardly affected realization, postponement, or abandonment of short-term childbearing intention.

Furthermore, age, partnership context, and parity turn out to be crucial for realizing short-term fertility intentions. Especially at later reproductive age childbearing intentions were significantly less often realized. Persons in their twenties (particularly in their late twenties) both realized and postponed their intentions to a higher extent while abandonment is less frequent in this age group. Living apart together with the partner is a less favourable context for realizing fertility intentions. Also persons without a partnership at wave 1 rarely realized their short-term intentions but often postponed or abandoned them. Finally, parity matters for realization. On average, childless persons more often realized or postponed their intentions. They, however, did not abandon them as likely as parents did.

The most interesting result, however, refers to education. Our results suggest that both, less educated as well as highly educated people are more likely to realize their childbearing intentions. However, while less educated were less likely to postpone, highly educated were less likely to abandon existing intentions.

Multilevel models

Table A5 presents multilevel logistic regression models with alternative model specifications (two-level random intercept and random coefficient models as well as a three-level random intercept model). Multilevel modelling allows accounting for variation in realization and

postponement between countries, including the variation of urban-rural differences across countries. Although eleven countries are not enough to follow a rigorous multilevel approach (see below), it is worth to study whether the differences between rural and urban regions are also found in multilevel models.

All models in Table A5 control for socio-demographic characteristics. Irrespective of concrete model specifications, multilevel models confirm the results that realization is lower and postponement is higher in urban than in rural regions. In case of realization, variation of urban-rural differences across countries seems to be negligible (Models ML2 and ML3 in Table A5). In case of postponement, however, some variation is revealed. Compared to regional variation in realization and postponement across countries, variation between urban and rural regions seem to be small (Model ML4).

The role of contextual variables

As eleven countries are not sufficient to prove contextual effects in multilevel models (the literature recommends 20 to 30 countries minimum), our research strategy comprised three different approaches to explore contextual effects:

(1) We correlate the urban-rural differences in realization and postponement on the country level with urban-rural differences in contextual factors. Correlations refer to ten or eleven countries, respectively, as some indicators are missing for single countries (Table A7).

(2) We conduct binomial and multinomial logistic regression models with country dummies for country fixed effects and corrected standard errors for context characteristics. Context characteristics are considered for 22 regions: one urban and one rural region for each of the eleven countries under study. We aim to find out

whether urban-rural differences are smaller in models including these context characteristics (Table A8).

(3) We analyse realization, postponement, and abandonment in three separate binomial logistic multilevel models accounting for 22 regions (Table A9). Instead of dummy variables for both the urban-rural difference and countries, these models include a regional random effect. If variation of contextual factors is responsible for differences between regions, we should detect a considerable reduction in variance across regions in models including context covariates.

In step 1 and step 2, we include all contextual variables, which means that a part of the analyses is restricted to 10 countries or 20 regions, respectively. The multilevel model in step 3 considers only the four contextual variables that are available for all eleven countries under study. All logistic regression models in step 2 and step 3 additionally control for socio-demographic characteristics. Unfortunately, none of the three approaches can fully account for both the complex structure of our data and the specific aim of our research, which focuses on variation in urban-rural differences in realization, postponement, and abandonment across countries (and *not* only on differences in realization, postponement, and abandonment between countries). The main shortcoming is that analyses of step 2 and step 3, which distinguish between 22 regions, do not consider that two regions always belong to the same country. This, however, may be crucial in an analysis of context effects.

Ad (1): The main findings on macro level associations regarding realization and postponement (medium to strong correlations; Table A7) can be summarised as follows: Urban-rural differences in indicators for educational and labor market opportunities, individuals' economic situation, and childbearing norms are linked with regional differences in realization of fertility intentions. The urban-rural difference in realization of short term fertility intentions is the larger, the larger urban-rural differences regarding people with economic difficulties and childlessness among women aged 40-45, and the smaller urban-

rural differences regarding high skilled professional occupations. Moreover, urban-rural differences in educational and labor market opportunities are related with postponement of fertility intentions: The larger urban-rural differences in the share of highly educated persons as well as in the share of high skilled professional occupations, the larger urban-rural differences in postponement. These findings suggest that better educational and economic opportunities lead to higher postponement in cities. At the same time, highly developed urban economies may also contribute to reduce realization gaps. In most countries rural regions are (on average) poorer than urban regions. Our results indicate that urban-rural differences in realization are less pronounced if economic differences between urban and rural regions are smaller. Finally, higher levels of childlessness (women age 40-45) in cities go hand in hand with lower realization among persons (age 18-45) who want a child.

Ad (2): Table A8 presents the results of five models. Model M4 (incorporated in Table 3 in the main text) includes the four contextual variables that are available for all eleven countries under study: use of childcare among children below age 3, mothers employed (age 25-45), the share of highly educated (age 25-45), and the share of childless women age 40-45. Model M4a additionally includes the share of people with difficulties to make ends meet. Compared to Model M4, models M4b to M4d each comprise one different indicator either for maternal employment, labour market opportunities, or norms/family views.

Differences between urban and rural regions are no longer statistically significant, when contextual variables are added to these models. This indicates that context factors explain urban-rural differences in realization, postponement, and abandonment.

Results further suggest that better childcare and job opportunities for mothers lead to higher realization and less postponement, but also to higher abandonment of intentions (Models M4 to M4d). The reason for the latter may be a culture that supports female careers. In line with this result, abandonment is higher if full-time work of mothers is more widespread (Model M4b). Better educational and labour market opportunities lead to lower

realization and more postponement (Models M4 to M4d), but do not contribute to higher abandonment. In addition, the higher the share of people with difficulties to make ends meet, the less likely are both realization and postponement, and the more likely is abandonment of fertility intentions (Model M4a).

Findings regarding norms are less straightforward. The relevance of motherhood in general does not seem to affect the realization of childbearing intentions (Model M4d). A higher degree of childlessness could even foster realization (Model M4a) and contribute to lower levels of postponement (Models M4 and M4c).

Ad (3): In sum, the findings depicted in Table A9 confirm the discussed results on contextual variables. In addition, they show that a substantial part of the regional variation in realization (44 %) and postponement (40 %) is explained by context covariates (compare regional SD of the reference model and of the full model). Likelihood-ratio tests confirm that models with contextual variables differ from the reference model without these variables. Overall, findings indicate that contextual effects contribute to variation in realization and postponement at the regional level.

Decomposition analyses

Table A10 provides details regarding models for country clusters that are discussed in the main text (Table 4). Tables A11 and A12 give results for single countries. Findings for single countries confirm that urban-rural differences in realization can be explained by differences in urban and rural populations in Western but not in Eastern Europe (Table A11; the only exceptions are Austria and Bulgaria). In addition, results for single countries emphasise once more that differences in partnership status between urban and rural populations are crucial for urban-rural differences in realization but differ between Eastern and Western Europe.

Figure A1. Fertility outcome and fertility intentions by countries

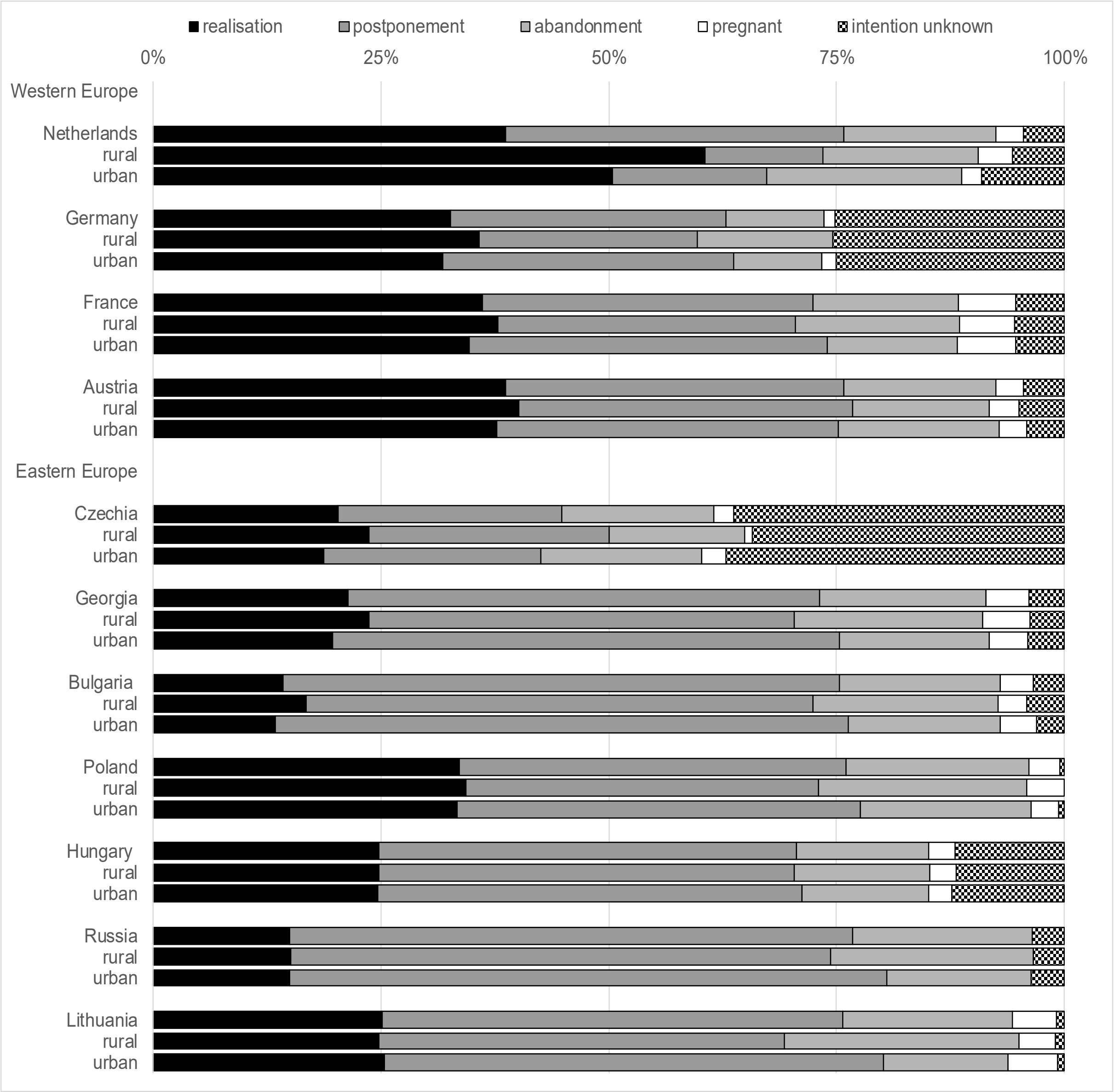

Source: GGS wave 1 and wave 2; panel respondents intending a child within three years in wave 1 (N = 11,329).

Figure A2. Urban-rural differences in realization of fertility intentions by countries (average marginal effects)

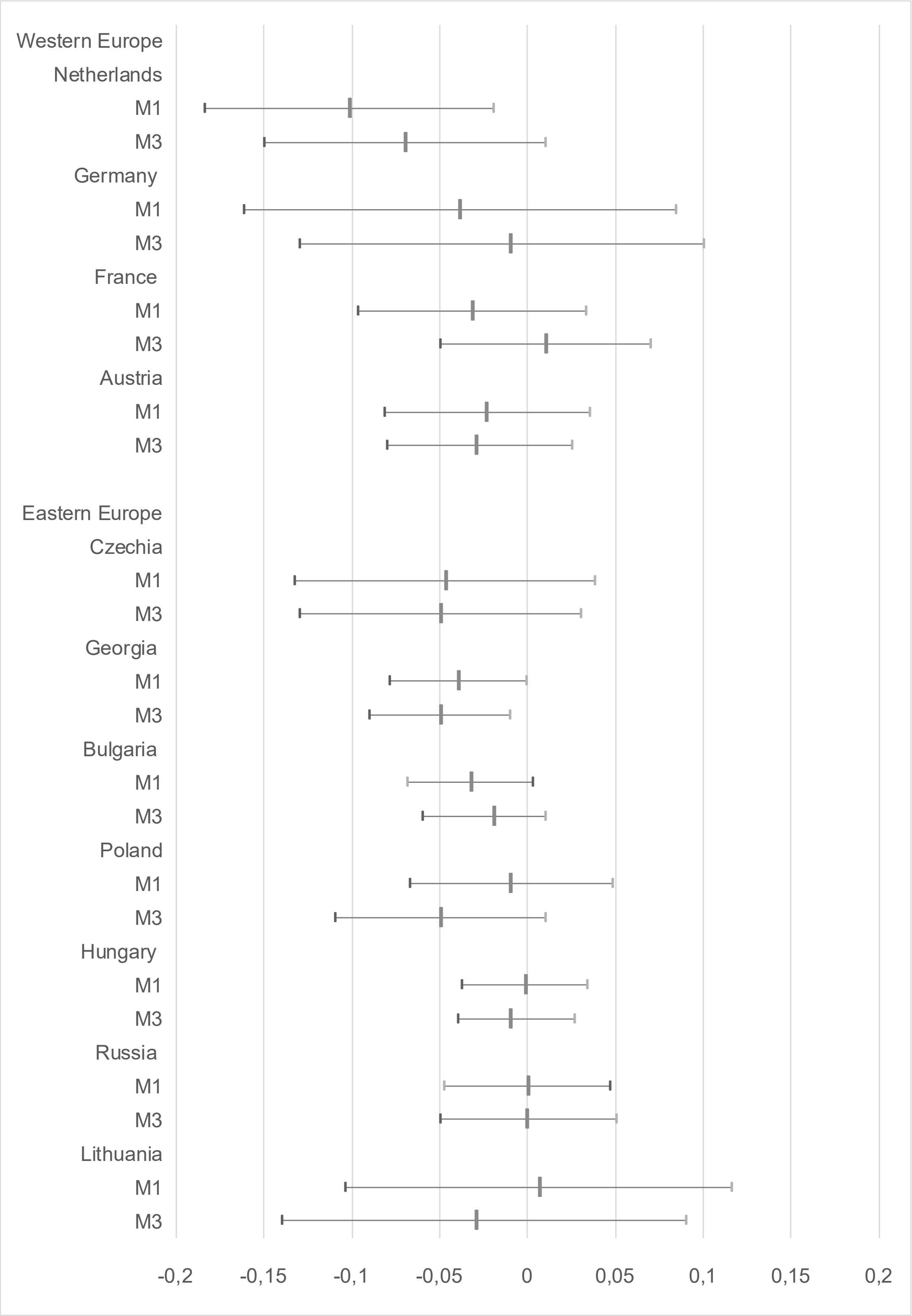

Note: The figure presents average marginal effects (AME) and corresponding 95% confidence intervals resulting from binary logistic regression models (model M1 without controls, M3 including controls; for details see method section).
Source: GGS wave 1 and wave 2; panel respondents intending a child within three years in wave 1.

Table A1. Differentiation between urban and rural areas by countries

Type of settlement		Rural	Urban
Austria	urban		X
	rural	X	
Bulgaria	urban		X
	rural	X	
Czechia	urban		X
	rural	X	
France	urban unity of Paris		X
	urban unity 200,000-1,999,999 inhabitants		X
	urban unity 100,000-199,999 inhabitants		X
	urban unity 50,000-99,999 inhabitants		X
	urban unity 20,000-49,999 inhabitants	X	
	urban unity 10,000-19,999 inhabitants	X	
	urban unity 5,000-9,999 inhabitants	X	
	urban unity with less than 5,000 inhabitants	X	
	rural community	X	
Georgia	capital		X
	urban		X
	rural	X	
Germany	central area >500,000 inhabitants		X
	peripheral area >500,000 inhabitants		X
	central area from 100,000 to 499,999 inhabitants		X
	peripheral area from 100,000 to 499,999 inhabitants		X
	central area from 50,000 to 99,999 inhabitants		X
	peripheral area from 50,000 to 99,999 inhabitants		X
	from 20,000 to 49,999 inhabitants	X	
	from 5,000 to 19,999 inhabitants	X	
	from 2,000 to 4,999 inhabitants	X	
	from 1 to 1,999 inhabitants	X	
Hungary	capital		X
	city		X
	town	X	
	village	X	
Lithuania	100,001 - 500,000 inhabitants		X
	50,001 - 100,000 inhabitants		X
	10,001 - 50,000 inhabitants	X	
	2,001 - 10,000 inhabitants	X	
	up to 2,000 inhabitants	X	
Netherlands	very strongly urbanised (2,500 or more addresses/km²)		X
	strongly urbanised (1,500 to less than 2,500 addresses/km²)		X
	moderately urbanised (1,000 to less than 1,500 addresses/km²)	X	
	hardly urbanised (500 to less than 1,000 addresses/km²)	X	
	not urbanised (less than 500 addresses/km²)	X	
Poland	capital		X
	urban		X
	rural	X	
Russia	oblast center		X
	town	X	
	urban-type community	X	
	rural area	X	

Table A2. Sample sizes for multivariate analyses

	(a) Realization (dichotomous)			*(b) Realization/ Postponement/ Abandonment*		
Country	Total	Urban	Rural	Total	Urban	Rural
Western Europe						
Austria	1,110	671	439	1,026	623	403
France	836	453	383	739	400	339
Germany	334	267	67	246	196	50
Netherlands	540	276	264	484	245	239
	2,820	*1,667*	*1,153*	*2,495*	*1,464*	*1,031*
Eastern Europe						
Bulgaria	1,700	1,243	457	1,580	1,156	424
Czechia	375	261	114	231	157	74
Georgia	1,685	946	739	1,541	868	673
Hungary	2,420	853	1,567	2,061	726	1,335
Lithuania	247	146	101	233	137	96
Poland	1,147	762	385	1,103	734	369
Russia	925	360	565	893	347	546
	8,499	*4,571*	*3,928*	*7,642*	*4,125*	*3,517*
Total	*11,319*	*6,238*	*5,081*	*10,137*	*5,589*	*4,548*

Note: Out of the 11,329 panel respondents, 10 were dropped due to missings in control variables.
Source: GGS wave 1 and wave 2; panel respondents intending a child within three years in wave 1.

Table A3. Logistic regression analysis with the pooled sample

Model:	M1 AME		M2 AME		M3 AME	
Type of settlement						
Rural (reference)	0		0		0	
Urban	-.02	**	-.03	**	-.03	***
Country						
Western Europe						
Austria			.25	***	.24	***
France			.21	***	.20	***
Germany			.18	***	.19	***
Netherlands			.40	***	.32	***
Eastern Europe						
Bulgaria (reference)			0		0	
Czechia			.06	*	.04	(*)
Georgia			.07	***	.10	***
Hungary			.11	***	.11	***
Lithuania			.10	***	.06	*
Poland			.21	***	.16	***
Russia			.00		-.02	
Timespan between Time 1 and Time 2						
Up to 48 months			.00		.00	
49-60 months (reference)			0		0	
61-72 months			-.02		-.01	
73-80 months			.00		.00	
Gender						
Male (reference)					0	
Female					-.01	
Age at Time 1						
18-24					.01	
25-29					.03	*
30-34 (reference)					0	
35-45					-.13	***
Partner status at Time 1						
Co-resident (reference)					0	
LAT					-.15	***
No partner					-.24	***
Parity at Time 1						
Childless (reference)					0	
1 child					.00	
2 children					-.10	***
3 or more children					-.04	
Education						
ISCED 0-2					.03	*
ISCED 3-4 (reference)					0	
ISCED 5-6					.03	***
Cragg-Uhler R^2	.00		.08		.19	

Note: (*) $p \leq .1$; * $p \leq .05$; ** $p \leq .01$; *** $p \leq .001$; N = 11,319.
Source: GGS wave 1 and wave 2; panel respondents intending a child within three years in wave 1.

Table A4. Multinomial logistic regression analysis with the pooled sample (model M3)

Model: M3	*Realized* AME		*Postponed* AME		*Abandoned* AME	
Type of settlement						
Rural (reference)	0		0		0	
Urban	-.03	***	.05	***	-.02	*
Country						
Western Europe						
Austria	.26	***	-.18	***	-.08	***
France	.24	***	-.16	***	-.08	***
Germany	.27	***	-.16	***	-.10	***
Netherlands	.38	***	-.38	***	.00	
Eastern Europe						
Bulgaria (reference)	0		0		0	
Czechia	.11	***	-.15	***	.04	
Georgia	.10	***	-.04		-.06	**
Hungary	.13	***	-.11	***	-.03	
Lithuania	.07	**	-.03		-.05	(*)
Poland	.16	***	-.15	***	-.02	
Russia	-.04	(*)	.10	***	-.06	**
Timespan between Time 1 and Time 2						
Up to 48 months	.01		-.02		.01	
49-60 months (reference)	0		0		0	
61-72 months	-.01		.00		.01	
73-80 months	-.01		.01		.00	
Gender						
Male (reference)	0		0		0	
Female	-.02	(*)	-.03	**	.04	***
Age at Time 1						
18-24	.02		.10	***	-.12	***
25-29	.04	**	.04	**	-.07	***
30-34 (reference)	0		0		0	
35-45	-.13	***	-.08	***	.21	***
Partner status at Time 1						
Co-resident (reference)	0		0		0	
LAT	-.16	***	.13	***	.02	(*)
No partner	-.25	***	.15	***	.10	***
Parity at Time 1						
Childless (reference)	0		0		0	
1 child	.00		-.13	***	.13	***
2 children	-.11	***	-.22	***	.32	***
3 or more children	-.02		-.20	***	.22	***
Education						
ISCED 0-2	.03	*	-.08	***	.05	***
ISCED 3-4 (reference)	0		0		0	
ISCED 5-6	.03	***	.02		-.05	***
Cragg-Uhler R^2					.35	

Note: (*) $p \leq .1$; * $p \leq .05$; ** $p \leq .01$; *** $p \leq .001$; N = 10,137.
Source: GGS wave 1 and wave 2; panel respondents intending a child within three years in wave 1.

Table A5. Multilevel models (alternative model specifications)

(a) Realization (dichotomous; N = 11,319)

Model	***ML 1***	***ML 2***	***ML 3***	***ML 4***
Levels	*2*	*2*	*2*	*3*
Fixed effects	*b*	*b*	*b*	*b*
Realization (dichotomous)				
Rural regions (reference)	0	0	0	
Urban regions	-.17***	-.17**	-.17**	
Constant	-.30	-.30	-.30	-.39*
Random effects	Est.SE	Est.SE	Est.SE	Est.SE
Country SD (constant)	.56(.12)	.56(.12)	.56(.13)	.55(.13)
Country SD (urban)		.06(.08)	.06(.08)	
Country r (urban, constant)			-.11(.94)	
Rural/Urban SD (constant)				.11(.05)
Chi² LR-Test (vs. non-ML)	398.25***	394.89***	394.91***	402.93***
Chi² LR-Test (vs. ML1)		-3.36		
Chi² LR-Test (vs. ML2)			.01	3.62(*)

(b) Postponement (dichotomous; N = 10,137)

Model	***ML 1***	***ML 2***	***ML 3***	***ML 4***
Levels	*2*	*2*	*2*	*3*
Fixed effects	*b*	*b*	*b*	*b*
Realization (dichotomous)				
Rural regions (reference)	0	0	0	
Urban regions	.22***	.22***	.21***	
Constant	-.19	-.19	-.19	-.07
Random effects	Est.SE	Est.SE	Est.SE	Est.SE
Country SD (constant)	.57(.13)	.56(.13)	.53(.12)	.56(.13)
Country SD (urban)		.12(.06)	.11(.06)	
Country r (urban, constant)			.58(.53)	
Rural/Urban SD (constant)				.16(.05)
Chi² LR-Test (vs. non-ML)	348.34***	349.53***	350.43***	358.57***
Chi² LR-Test (vs. ML1)		1.18		
Chi² LR-Test (vs. ML2)			.90	11.33***

Note: Timespan, gender, age, partner status, parity, and education are included as controls in all models.
(*) p ≤ .1; * p ≤ .05; ** p ≤ .01; *** p ≤ .001.
Source: GGS wave 1 and wave 2; panel respondents intending a child within three years in wave 1.

Table A6. Urban-rural differences in contextual variables by countries

Shares are given in %; differences in percentage points	Childcare		Maternal employment				Educational & Labour market opportunities				Economic situation		Norms/ traditional family views			
	among children below age 3		employed mothers		fulltime employed		highly educated (25-45)		ISCO 1-3 (25-45)		difficulties making ends meet		childless women age 40-45		women need child to be fulfilled	
	rural	urban (+/-)	rural	urban (+/-)	rural	urban (+/-)	rural	urban (+/-)	rural	urban (+/-)	rural	urban (+/-)	rural	urban (+/-)	rural	urban (+/-)
Western Europe																
Austria	33	+8	67	-2	21	+1	14	+11	33	+12	22	+7	12	+8	29	-4
France	52	+2	71	-2	45	+4	29	+15	34	+16	54	-1	10	+11	56	-3
Germany	42	-5	58	-2	26	-4	21	+7	/	/	39	1	10	+5	31	-2
Netherlands	42	+9	62	+1	9	+9	31	+14	56	+6	/	/	14	+11	/	/
Eastern Europe																
Bulgaria	28	-4	53	+19	46	+22	7	+25	15	+23	96	-6	6	+2	66	-14
Czechia	27	-7	62	-1	56	-2	15	+3	35	+9	64	-2	9	+3	69	-1
Georgia	4	+14	35	+2	19	+9	18	+28	22	+27	86	-9	14	-2	93	1
Hungary	37	+15	56	+15	50	+12	14	+20	28	+22	64	-12	5	+2	81	-1
Lithuania	25	+6	72	+7	63	+10	18	+23	31	+23	54	-13	9	+1	44	-1
Poland	20	+11	58	+7	/	/	17	+24	18	+24	57	-10	6	+7	49	-10
Russia	24	+4	73	+1	68	+2	39	+39	12	+32	90	-9	95	+4	84	-3

Source: GGS wave 1 (respondents age 18-45, 25-45 or 40-45).

Table A7. Correlation of urban-rural differences in contextual variables, realization and postponement

Urban-rural differences in	Realization	Postponement	(N)
Childcare opportunities:			
Use of childcare among children below age 3	.18	-.08	(11)
Maternal employment:			
Mothers employed (age 25-45)	.31	.04	(11)
Mothers fulltime employed (age 25-45)	.04	.17	(10)
Educational and labour market opportunities:			
Share of highly educated (ISCED 5-6) (age 25-45)	.28	**.42**	(11)
Share of high skilled professional occupations (ISCO 1-3) (age 25-45)	**.48**	**.51**	(10)
Economic situation:			
Share of people with difficulties making ends meet (age 18-45)	**-.60**	-.29	(10)
Norms/ family views:			
Share of childless women age 40-45	**-.41**	-.21	(11)
Share agreeing that women need child(ren) to be fulfilled (age 18-45)	.00	-.03	(10)

Note: Urban-rural differences in indicators were assessed using GGS data of wave 1. N refers to the number of countries with available information in wave 1. Realization (postponement) refers to the share of panel respondents intending a child within three years in wave 1 who realized their intention until wave 2 (who did not realize their intention but still uphold their intention at wave 2). For better visibility, correlation coefficients larger/smaller than +/- .4 are printed in bold type.
Source: GGS wave 1 and wave 2.

Table A8. Contextual variables and urban-rural differences in realization, postponement, and abandonment in logistic regression models with corrected standard errors

Logistic regression model:<br>Outcome:	(a) dichotomous<br>realization	realized	(b) multinomial<br>postponed	abandoned
**M4**	*AME*	*AME*	*AME*	*AME*
Rural regions/urban regions (0/1)	.00	.01	-.01	.00
Use of childcare among children below age 3	.12 **	.08	-.23 ***	.15 *
Mothers employed (age 25-45)	.17 ***	.21 ***	-.34 ***	.12 *
Share of highly educated (ISCED 5-6) (age 25-45)	-.28 ***	-.33 ***	.53 ***	-.21
Share of childless women age 40-45	.15	.05	-.21 *	.16
Cragg-Uhler R^2	.19		.35	
N	11,319		10,137	
**M4a**	*AME*	*AME*	*AME*	*AME*
Rural regions/urban regions (0/1)	.00	.01	-.02	.01
Use of childcare among children below age 3	.09 *	.05	-.31 ***	.26 ***
Mothers employed (age 25-45)	.09 *	.12 **	-.39 ***	.27 ***
Share of highly educated (ISCED 5-6) (age 25-45)	-.33 ***	-.40 ***	.55 ***	-.15
Share of childless women age 40-45	.35 **	.27 *	-.12	-.15
Share of people with difficulties making ends meet (age 18-45)	-.24 **	-.30 ***	-.22 ***	.52 ***
Cragg-Uhler R^2	.17		.33	
N	10,779		9,635	
**M4b**	*AME*	*AME*	*AME*	*AME*
Rural regions/urban regions (0/1)	.01	.02	-.04	.02
Use of childcare among children below age 3	.13*	.10	-.36***	.25**
Mothers fulltime employed (age 25-45)	.15	.22	-.57***	.35**
Share of highly educated (ISCED 5-6) (age 25-45)	-.33*	-.40*	.85***	-.45*
Share of childless women age 40-45	.06	-.03	-.07	.10
Cragg-Uhler R^2	.20		.35	
N	10,172		9,034	
**M4c**	*AME*	*AME*	*AME*	*AME*
Rural regions/urban regions (0/1)	-.02	-.01	.01	.00
Use of childcare among children below age 3	.10	.08	-.20**	.13$^{(*)}$
Mothers employed (age 25-45)	.15$^{(*)}$	.20*	-.30**	.10$^{(*)}$
Share of high skilled professional occupations (ISCO 1-3) (age 25-45)	-.17	-.23$^{(*)}$	.41**	-.18
Share of childless women age 40-45	.20	.12	-.29$^{(*)}$	.17
Cragg-Uhler R^2	.19		.35	
N	10,985		9,891	
**M4d**	*AME*	*AME*	*AME*	*AME*
Rural regions/urban regions (0/1)	.02	.03$^{(*)}$	-.03	.00
Use of childcare among children below age 3	.12$^{(*)}$	.06	-.28***	.23$^{(*)}$
Mothers employed (age 25-45)	.15**	.22***	-.29***	.07
Share of highly educated (ISCED 5-6) (age 25-45)	-.34***	-.36***	.62***	-.27*
Share agreeing that women need child(ren) to be fulfilled (age 18-45)	.00	.09	.13	-.22
Cragg-Uhler R^2	.17		.33	
N	10,779		9,653	

Note: Country, timespan, gender, age, partner status, parity, and education are included as control variables.
$^{(*)}$ $p \leq .1$; * $p \leq .05$; ** $p \leq .01$; *** $p \leq .001$.
Source: GGS wave 1 and wave 2; panel respondents intending a child within three years in wave 1.

Table A9. Contextual variables and urban-rural differences in realization, postponement, and abandonment in multilevel logistic regression with a regional random effect

Logistic multilevel regression models	Separate models with dichotomous variables		
Outcome:	realized	postponed	abandoned
**(a) Reference model**			
Fixed effects:	*b*	*b*	*b*
Constant	-.37 **	-.08	-2.67 ***
Random effect:	*Est. SE*	*Est. SE*	*Est. SE*
Regional SD (constant)	.55 (.09)	.57 (.09)	.29 (.06)
**(b) Model with contextual variables**			
Fixed effects	*b*	*b*	*b*
Use of childcare among children below age 3	2.50 ***	-2.46 **	-.32
Mothers employed (age 25-45)	-1.46 (*)	.95	.25
Share of highly educated (ISCED 5-6) (age 25-45)	-1.50 *	2.06 **	-.83
Share of childless women age 40-45	5.40 **	-5.39 **	-.58
Constant	-.50	.45	-2.41 ***
Random effects	*Est. SE*	*Est. SE*	*Est. SE*
Regional SD (constant)	.31 (.06)	.34 (.06)	.26 (.06)
Chi² LR-Test (model a vs. b)	22.70 ***	20.59 ***	3.49
$N_{individuals}$	11,319	10,137	10,137
$N_{regions}$	22	22	22

Note: A constant as well as timespan, gender, age, partner status, parity, and education (as control variables) have been included in both models. (*) p ≤ .1; * p ≤ .05; ** p ≤ .01; *** p ≤ .001.
Source: GGS wave 1 and wave 2; panel respondents intending a child within three years in wave 1.

Table A10. Detailed results of decomposition analyses for country clusters

Decomposition	Realization (dichotomous) Western Europe	Realization (dichotomous) Eastern Europe	Postponement (dichotomous) Western Europe	Postponement (dichotomous) Eastern Europe
Gender				
Male (reference)	0	0	0	0
Female	.000	.001	.000	.002 **
Age at Time 1				
18-24	.000	.001	.002 **	.004 ***
25-29	.000	.000	.000	.000
30-34 (reference)	0	0	0	0
35-45	.010 ***	.001	.001	.001 **
Partner status at Time 1				
Co-resident with partner (reference)	0	0	0	0
LAT	-005 ***	.003 ***	-.005 ***	-.004 ***
No partner	.019 ***	-.007 ***	-.018 ***	.005 ***
Parity at Time 1				
Childless (reference)	0	0	0	0
One child	.001 *	.001 **	-.002	.002 ***
2 or more children	-.002	-.004 ***	-.005 ***	-.006 ***
Education				
ISCED 0-2	.000	.002	.000	-.005 ***
ISCED 3-4 (reference)	0	0	0	0
ISCED 5-6	-.005 **	-.004 (*)	.003 (*)	-.010 ***

Note: Decomposition analyses shown refer to model M3 without timespan and country. Our conclusions are not altered if these variables are additionally included (but the sum of effects in Table A6 would not correspond to coefficients in Table 4). (*) $p \leq .1$; * $p \leq .05$; ** $p \leq .01$; *** $p \leq .001$.
Source: GGS wave 1 and wave 2; panel respondents intending a child within three years in wave 1.

Table A11. Results of decomposition analyses for single countries

**Country**	Probability of realization Rural	Urban	Difference in realization	Explained by composition
Western Europe				
Austria	.401	.378	.024	-.004
France	.379	.347	.032	.041
Germany	.358	.318	.040	.029
Netherlands	.606	.504	.102	.039
Eastern Europe				
Bulgaria	.168	.134	.035	.013
Czechia	.237	.188	.049	.003
Georgia	.237	.197	.040	-.007
Hungary	.248	.246	.002	-.006
Lithuania	.248	.253	-.006	-.026
Poland	.343	.333	.010	-.032
Russia	.150	.150	.000	-.001

Source: GGS wave 1 and wave 2; panel respondents intending a child within three years in wave 1.

Table A12. Detailed results of decomposition analyses for selected countries

Decomposition	France	Germany	Netherlands	Bulgaria	Georgia
Gender					
Male (reference)	0	0	0	0	0
Female	.000	.006	-.005	.000	.002 (*)
Age at Time 1					
18-24	.000	.000	.001	-.003	.007 ***
25-29	.001	.005	.001	-.001	-.004 *
30-34 (reference)	0	0	0	0	0
35-45	.005 *	.001	.016 ***	.002	.001
Partner status at Time 1					
Co-resident with partner (reference)	0	0	0	0	0
LAT	.007 **	-.001	.003	.006 **	.003 *
No partner	.033 ***	.014 **	.036 ***	-.011 ***	-.020 ***
Parity at Time 1					
Childless (reference)	0	0	0	0	0
One child	-.001	-.002	.003	.003 (*)	.009 *
2 or more children	.000	-.003	-.003	-.004 *	-.010 *
Education					
ISCED 0-2	.000	.005	.000	.016 *	.002
ISCED 3-4 (reference)	0	0	0	0	0
ISCED 5-6	-.004	-.001	-.013 *	.004	.003

Note: (*) p ≤ .1; * p ≤ .05; ** p ≤ .01; *** p ≤ .001.
Source: GGS wave 1 and wave 2; panel respondents intending a child within three years in wave 1.
